# Conservation of location of several specific inhibitory codon pairs in the *Saccharomyces sensu stricto yeasts* reveals translational selection

**DOI:** 10.1093/nar/gky1262

**Published:** 2018-12-21

**Authors:** Dalia H Ghoneim, Xiaoju Zhang, Christina E Brule, David H Mathews, Elizabeth J Grayhack

**Affiliations:** 1 Department of Biochemistry and Biophysics, School of Medicine and Dentistry, University of Rochester, Rochester, NY 14642, USA; 2 Center for RNA Biology, University of Rochester, Rochester, NY 14642, USA

## Abstract

Synonymous codons provide redundancy in the genetic code that influences translation rates in many organisms, in which overall codon use is driven by selection for optimal codons. It is unresolved if or to what extent translational selection drives use of suboptimal codons or codon pairs. In *Saccharomyces cerevisiae*, 17 specific inhibitory codon pairs, each comprised of adjacent suboptimal codons, inhibit translation efficiency in a manner distinct from their constituent codons, and many are translated slowly in native genes. We show here that selection operates within S*accharomyces sensu stricto* yeasts to conserve nine of these codon pairs at defined positions in genes. Conservation of these inhibitory codon pairs is significantly greater than expected, relative to conservation of their constituent codons, with seven pairs more highly conserved than any other synonymous pair. Conservation is strongly correlated with slow translation of the pairs. Conservation of suboptimal codon pairs extends to two related *Candida* species, fungi that diverged from *Saccharomyces* ∼270 million years ago, with an enrichment for codons decoded by I•A and U•G wobble in both *Candida* and *Saccharomyces*. Thus, conservation of inhibitory codon pairs strongly implies selection for slow translation at particular gene locations, executed by suboptimal codon pairs.

## INTRODUCTION

Synonymous codons specify insertion of the same amino acid into the nascent polypeptide, a redundancy in the genetic code that provides an opportunity for fine-tuned regulation of translation while preserving the resulting protein sequence (1). Synonymous codons differ in their use between genomes and within individual genes in a single genome, in the abundance of the tRNAs available to decode them, in the necessity for wobble interactions to decode them and in the accuracy and speed with which they are decoded (2–7). Synonymous codons also differ in their preference for neighboring codons, resulting in a non-random distribution of codon pairs (combinations of adjacent codons in a particular order) across all domains of life (8,9). The analysis of codon usage and its conservation has yielded information on some of the functionally important roles of codon choice in translation. However, it has been difficult to resolve the full spectrum of translational selection on codon use, in part because the forces that drive the selection of synonymous codons within a genome are thought to be split among selection for translation, selection for mRNA features (such as splicing or secondary structure) and mutational drift (10,11).

There is a compelling case that, in many organisms, selection for rapid and/or accurate translation (12–16) drives the overuse of a genome-specific subset of codons, called optimal codons. The optimal codons are generally those decoded by the most abundant tRNAs (17,18). In these organisms, optimal codons are used at higher than expected frequencies throughout the genome and in even greater proportions in highly expressed genes. The prevailing idea is that translational selection operates more robustly on highly expressed genes than on poorly expressed genes, because alterations in translation of highly expressed genes will have a larger impact on the global rate of protein synthesis, which is limited by the pool of free ribosomes (6,10). Consistent with this idea, highly expressed genes evolve at the slowest rates, both with respect to amino acid and synonymous codon changes (13,19).

By contrast, the translational importance of suboptimal codons remains puzzling, in part because suboptimal codons are depleted in highly expressed genes, while poorly expressed genes evolve rapidly (20–22), making it difficult to assess selection by conservation of suboptimal codons. Moreover, codon pairs can modulate both translation efficiency and elongation in a manner distinct from their constituent codons (23–26), further complicating suboptimal codon effects. The persistence of suboptimal codons has been proposed to be the result of weaker selection for expression of poorly expressed genes, whose composition is instead shaped by neutral processes such as biased spontaneous mutation (27), GC-biased gene conversion during meiotic recombination (28) and neutral genetic drift (10,29,30).

Two types of arguments have been advanced to assert that suboptimal codons play important roles in translation. First, evolutionary signatures of rare codon selection across species provides evidence of translational selection of rare codons. Rare codons are posited to be slowly translated, suboptimal codons. In a wide range of organisms from bacteria to humans, rare codons frequently occur in clusters within a local region of a gene, contrary to the expectation for neutral genetic drift (31,32). Furthermore, these clusters are frequently conserved in homologous genes across distantly related species (33), and in *Escherichia coli* are often found in locations associated with co-translational folding intermediates (34). Second, codon-mediated effects on protein folding have been demonstrated directly using real-time FRET (35), and inferred from effects of a synonymous change in the *Multidrug Resistance 1* gene that alters the specificity of P glycoprotein, affecting a significant fraction of the population (36). Furthermore, replacing suboptimal codons with synonymous optimal codons altered function of circadian regulators in both *Neurospora* and cyanobacteria (37,38). These results support the long-standing idea that the slow translation of ribosomes through suboptimal codons modulates correct folding of the nascent polypeptides (39,40). However, the issue of translational importance of suboptimal codons has remained controversial because there is no direct evidence of selection of known suboptimal codons.

We recently identified 17 specific inhibitory codon pairs (ICPs) in the yeast *Saccharomyces cerevisiae* that inhibit protein expression substantially more than their individual constituent codons and are each composed of adjacent suboptimal codons (25). These ICPs were identified among the 3,721 possible codon pairs by analysis of libraries of GFP variants in which three codons in frame with GFP were randomized. Expression of GFP variants was quantified by fluorescence-activated cell sorting (FACS) followed by deep sequencing to obtain a score for each nine nucleotide variant (GFP^SEQ^), which was then normalized to the most highly expressed synonymous variant (syn-GFP^SEQ^). ICPs were distinguished based on their enrichment in poorly expressed GFP variants, with their syn-GFP^SEQ^ median scores ranging from 0.44 to 0.82. Inhibitory effects of these pairs are generally caused by defects in translation, because, for 11 of 12 pairs tested, expression of particular tRNAs suppressed their inhibitory effects (25). Many of these ICPs, which occur in 1868 of 5917 *S. cerevisiae* genes, are translated very slowly in native yeast genes, based on ribosome density measurements. These *S. cerevisiae* ICPs lose their inhibitory effect when the codons in the pair are separated, reversed, or out of frame, and no individual codon can explain inhibition (25). Moreover, these ICPs are enriched in codons decoded by I•A or U•G wobble decoding, and in codons with the lowest metrics for translation selection in multiple indexes (3,15,41,42). If the impact of these ICPs on translation is functionally important, then selective pressure on some or all of these 17 codon pairs might differ from that on other codon pairs or on their constituent codons.

We evaluated the position-specific conservation of individual codons and codon pairs in coding regions of closely related fungi, first across five species of *Saccharomyces sensu stricto* yeasts (closely related to *S. cerevisiae)*, and then between two related species in the *Candida* clade, which diverged from *Saccharomyces sensu stricto* yeasts ∼270 million years ago. In both sets of organisms, 32–40 codon pairs of 3,721 are remarkably more highly conserved than expected based on the conservation of their constituent codons, although in general there is a strong overall correlation between the conservation of individual codons and the conservation of codon pairs made up of those codons. In the *Saccharomyces sensu stricto* yeasts, these highly conserved pairs include nine of the 17 ICPs (compared to 31 of 3704 other codon pairs); these nine ICPs are also the most slowly translated pairs in the yeast genome. In the *Candida* clade, the highly conserved codon pairs include pairs that, like those in *S. cerevisiae*, are composed of codons decoded by I•A or U•G wobble. Conservation of slowly translated ICPs provides evidence of their functional importance and of translational selection operating on suboptimal codon use, a mechanism for gene regulation that appears to be conserved in the broad spectrum of Saccharomycotina fungi (43).

## MATERIALS AND METHODS

### *Saccharomyces sensu stricto* ORF sequence and ortholog assignment

Coding ORF sequences of *S. cerevisiae* (UTRs, introns, and untranslated bases removed) were obtained from *Saccharomyces* Genome Database (SGD) (44). Dubious ORFs and pseudogenes were excluded from our analysis.

A publicly available dataset of 5261 orthologs was used for the analysis of *Saccharomyces sensu stricto* (45). *Saccharomyces sensu stricto* multi-organism alignments (*S. bayanus* var. *uvarum, S. kudriavzevii, S. mikatae, S. cerevisiae* and *S. paradoxus)* were produced as described in the ‘Ortholog alignment’ section of methods. For the five-species alignments, the dataset was modified to include only genes that have orthologs in all five species (nine ORFs did not include all five species). For pairwise species comparisons, genes with alignments across two species were included. We performed revisions to the original dataset: First, 35 of the ortholog alignment files had redundant orthogroup assignments. To resolve the identity of each of these genes, we used BLASTN 2.0 (46) on *S. cerevisiae* reference sequence and synteny of chromosomal coordinates. Nineteen of these redundant genes were found to have one alignment that maps to the gene and the other alignment was reassigned to the paralog of that gene. Eleven genes had two sequence files mapping to the same gene with BLAST. Of these, six genes contained an intron resulting in one full-length sequence and one sequence truncated near the site of the intron. In other cases, no intron was present, but the gene still had one full-length sequence and one truncated sequence. We only considered the full-length *S. cerevisiae* sequences. Second, one ORF (YOR239W) contains a known +1 frameshift and was recorded in the dataset as two partial transcript sequences with different reading frames. These two sequences were concatenated to form one alignment with one adenosine residue removed to maintain the correct frame. Finally, there were 35 ORFs in the dataset with no assigned SGD identities. BLASTN 2.0 was used to determine the identity of these ORFs. Our final dataset consists of 5161 alignments out of 5917 total *S. cerevisiae* genes.

### *Candida* ORF sequences and ortholog assignment

*Candida albicans* SC5314 reference genome was sequenced by the Stanford Genome Technology Center (47) and the Biotechnology Research Institute of the National Research Council of Canada (48). We used the publicly available file ‘C_albicans_SC5314_A22_current_chromosomes.fasta.gz’ (updated 22 November 2015) from the Candida genome database (CGD) (49) that contains sequences for 12421 coding ORFs with introns and untranslated sequences removed. Because of the obligate diploid nature of *Candida albicans*, only one allele was used in our analysis and the ‘A’ allele was arbitrarily selected. Dubious genes and pseudogenes were excluded resulting in a total of 6041 ORFs in our analysis.

*Candida dubliniensis* CD36 genome was sequenced by the Wellcome Trust Sanger Institute (50). The sequence file ‘C_dubliniensis_CD36_current_chromosomes.fasta.gz’ with introns and untranslated sequences removed was obtained from the CGD (updated 21 June 2015).

We identified orthologous ORFs across the *Candida* species using the ‘Pillars.tab’ file (updated 7 February 2013) available from the Candida Gene Order Browser, version 2 (51,52). This table of orthologs across yeast species is based on reciprocal best matches as well as manual curation for accurate classification of orthologs. There are a total of 5516 orthologs shared across the two species. We constructed multi-species alignment files for each ortholog as described in the ‘Ortholog alignment’ methods section. We used a total of 5789 orthologs in our *Candida* analysis after exclusion of dubious ORFs and pseudogenes.

### Ortholog alignment

We determined orthologs for multiple sequence alignments from the publicly available ‘Pillars.tab’ list of orthologs from the Yeast Gene Order Browser (53). To achieve accurate alignments, we aligned the amino acid sequences of orthologous genes. Nucleotide sequences were translated to amino acid sequences using the Biopython, version 1.65, package Bio.Seq (54). The Standard Code (translation table 1) was used for translation of *Saccharomyces sensu stricto* sequences and the Alternative Yeast Nuclear Code (translation table 12) for translation of *Candida* sequences. We aligned the resulting amino acid sequences with the MUSCLE, version 3.8.31 (55) multiple sequence alignment tool using default settings. Sequences were then reverted back to their nucleotide sequences for our analysis.

### Whole coding-sequence-wide codon pair conservation score

We set up a scoring system to quantify the conservation of each codon and codon pair. One species in each set of alignments was assigned as a reference*. S. cerevisiae* was the reference in all the alignments in which it appears, *S. kudriavzevii* was the reference for the *S. kudriavzevii* to *S. mikatae* comparison, and *C. albicans* was selected as a reference in the *Candida* comparison. We computed the conservation rates of all nonterminating codon and codon pairs by determining the proportion with which each codon or codon pair is conserved to the number of times it appears in the reference (see Equations 1 and 2 in Results). A conserved codon or conserved codon pair was defined as having an identical sequence and location in the alignment across all species. The normalized conservation score (see Equation 3 in Results) quantifies the codon pair conservation of each codon pair relative to the product of the codon conservation rates of its constituent codons.

### Linear model and outliers

A line was fit to estimate the codon pair conservation rate of each of 3721 codon pairs as a function of the product of codon conservation rates of the constituent codons. The linear model function in R version 3.1.3 (56) was used to determine the best fit line with y-intercept set to zero. *Q*–*Q* plots were used to determine that the distributions of conservation rates are not skewed to either side although we find that the tails of the distributions are heavier than a normal distribution, and in particular the right tail. Pseudocounts were added in the calculation of the normalized conservation rate. Outliers to the model were defined as codon pairs with normalized conservation scores greater than three standard deviations from the best fit line.

### *z*-score distribution plots

*z*-score is a measure of the distance of a sample from the mean in units of standard deviation (σ). *z*-scores of the log transformed normalized conservation scores for each group of codon pairs encoding the same dipeptide were calculated, assuming a normal distribution.

Pseudocounts were introduced to handle conservation values of zero. The pseudocount value was added to the codon pair conservation rate and to each of the codon conservation rates in Equation 3 in Results. The value of pseudocounts was the reciprocal of the number of codons in the *S. cerevisiae* reference coding region as this represents the minimum level of variance that we could observe in our alignments.

### Whole-ORF codon conservation rate

For each gene, the ORF codon conservation rate is the fraction of codons in a given ORF that are conserved in all five species. Because the length of a gene can vary across species, we selected one species in each comparison to be the reference species for gene length. For the codon conservation rate in the *Saccharomyces sensu stricto* yeasts, the lengths of *S. cerevisiae* genes were used as the reference in the five species analysis and all pairwise analysis.

### Location of ICP_cons_ in ORFs

To assess whether there is a location bias for ICP_cons_ in certain regions or termini of ORFs, we compared the positions of ICP_cons_ in ORFs to the positions of 100 randomly selected sets of nine codon pairs. We calculated the median positions for each set in the 100 sets of randomly chosen codon pairs. Then, for the set of the median positions, we calculated the mean and standard deviation.

### Gene properties analysis

We explored properties of sets of genes to understand the features of genes that contain conserved inhibitory codon pairs. The CAI, Codon Bias, length, and molecular weight data were obtained from the ‘protein_properties.tab’ file available on *Saccharomyces* Genome Database (updated 26 January 2015) (44). The protein abundance was determined from publicly available Tap-tag and Mass Spectrometry data obtained from Ghaemmaghami *et al.* (57) and Kulak *et al.* (58), respectively. mRNA half-life data was obtained from Peccarelli and Kebaara (59). Kolmogorov-Smirnov tests on each pair of distributions were used to test the null hypothesis that the distributions are the same and are reported in Table 1. The value of α was set to 0.05. Only the intersection of 5161 genes in our dataset and genes in each of the mentioned files was used in each test.

**Table 1.** Properties of *S. cerevisiae* ORFs with conserved ICP_cons_, with conserved ACPs, and all other ORFs

	All ORFs^d^		ICP_cons_ ORFs^e^		ACP ORFs^f^		*P*-value^g^		
	Median	Mean	Median	Mean	Median	Mean	All; ICP_cons_	All; ACP	ICP_cons_; ACP
**CAI**	0.14	0.187	0.14	0.137	0.13	0.127	2.20 × 10^−16^*	4.74 × 10^−9^*	5.14 × 10^−4^*
**ORF length (codons)**	403	484.6	528	633.3	533	678.3	7.48 × 10^−10^*	2.63 × 10^−7^*	0.791
**Protein molecules per cell-TAP^a^**	2400	12580	1070	2230	2780	7164	2.20 × 10^−16^*	0.134	3.45 × 10^−10^*
**Protein molecules per cell-mass spec^b^**	926.2	10170	177	435.3	835.1	9748	2.20 × 10^−16^*	0.734	1.23 × 10^−14^*
**mRNA half life^c^**	7.4	11.73	6.4	8.396	7.3	10.34	5.43 × 10^−5^*	0.496	0.0723

^a^(57).

^b^(58).

^c^(59).

^d^ORFs that are neither in ICP_cons_ or ACP subsets.

^e^ORFs containing one or more ICP_cons_ conserved across at least four species of *Saccharomyces sensu stricto* yeasts.

^f^ORFs containing one or more ACP that is conserved across at least 4 species of *Saccharomyces sensu stricto* yeasts.

^g^ *P*-value determined using Kolmogorov Smirnov test on the distribution of values across two sets of ORFs (*x*; *y*). Asterisk (*) indicates significance with α = 0.05.

We obtained data regarding categorization of *S. cerevisiae* genes across monosomes and polysomes from Heyer and Moore (60). We looked at the proportion of genes in each of the five categories in Heyer's dataset (ORF < 590, monosome, no enrichment, polysome and polysome top 300) and used a chi-squared test to test the null hypothesis that the distribution for each of the categories is the same. We set α to 0.05.

### GO analysis

Gene Ontology enrichment analysis was performed using GoTermFinder version 0.83 hosted on the SGD webserver. We set α to 0.01, and as a background set, we used ORFs only, eliminating dubious ORFs. Only manually curated and high throughput annotations were considered.

### Biopython, R

Scripts were written in python and used the Biopython module to parse and translate fasta and alignment files. Graphs and plots were created in R.

## RESULTS

### Nine ICPs are highly conserved across *Saccharomyces sensu stricto* yeasts

**Figure 1.**
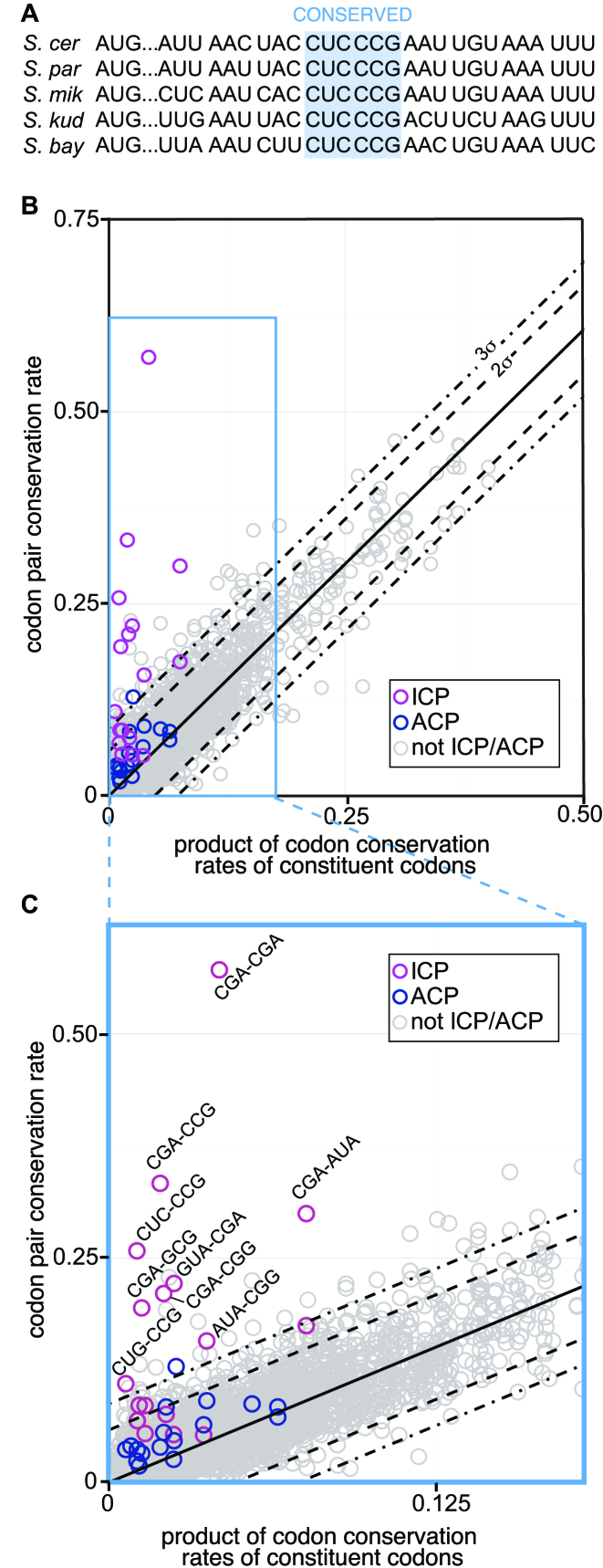
Nine ICPs are highly conserved across five *Saccharomyces sensu stricto* yeasts. (**A**) Schematic of aligned ORFs illustrating a conserved codon pair. Conservation was defined as identity across all five species. (**B**) Codon pair conservation rate plotted as a function of the product of conservation rates of its constituent codons. The solid line represents the best-fit line (slope = 1.21). Codon pairs with values >3σ from the line were considered outliers. Two sets of codon pairs are highlighted in the plot: ICPs (magenta) and ACPs (blue), a control set of codon pairs formed either by reversal of the codons in the ICPs or by use of codons with a similar CAI value when ICP reversal resulted in another ICP. (**C**) Inset of Figure 1B showing identity of ICP outliers.

To examine conservation of codons and codon pairs in the *Saccharomyces sensu stricto* yeasts, we aligned 5161 *S. cerevisiae* open reading frames (ORFs) with their orthologs (45) across four other *Saccharomyces sensu stricto* yeast species: *S. paradoxus, S. mikatae, S. kudriavzevii*, and *S. bayanus* var. *uvarum*. To specifically examine the subset of positions at which a particular codon or codon pair is strongly selected, we counted a codon or codon pair as conserved if and only if it aligned at the same position in the alignment in each of these ORFs across all five *Saccharomyces* *sensu stricto* organisms (Figure 1A). The codon and codon pair conservation rates of each of the 61 non-terminating codons and 3721 non-terminating codon pairs (Supplementary Table S1) were calculated across the set of multiple sequence alignments with *S. cerevisiae* as the reference:

(1) \begin{eqnarray*}codon\ conservation\ rate\ = \ \frac{{Total\ conserved\ codon\ counts}}{{Total\ codon\ counts\ in\ reference}}\end{eqnarray*}

(2) \begin{eqnarray*}&&codon\ pair\ conservation\ rate\ = \nonumber\\ && \frac{{Total\ conserved\ codon\ pair\ counts}}{{Total\ codon\ pair\ counts\ in\ reference}}\end{eqnarray*}

In comparing the codon conservation rates of individual codons across the five species, we found a wide range of conservation rates from 0.061 (Ala GCG) to 0.424 (Arg CGU) among codon families with two to six synonymous codons (Supplementary Figure S1), with even greater codon conservation rates for the single codon families (0.945 Trp UGG and 0.732 Met AUG). The wide range of conservation rates within synonymous codon and codon pair families (Supplementary Figure S1 and Supplementary Table S1) implies that there are differences in selective pressures related to codon use, an important consideration for a robust analysis of codon conservation. One might expect that codon conservation rates would correlate with codon optimality, since optimal codon use is driven by translational selection and is enriched in highly expressed genes, which are themselves more conserved than poorly expressed genes. Surprisingly, codon conservation rates were only weakly correlated with the Codon Adaptation Index (CAI) (41), the tRNA Adaptation Index (tAI) (3), and a selection coefficient based on gene expression levels in *S. cerevisiae* (15) (Supplementary Figure S1) (linear *r*^2^ of 0.19, 0.05 and 0.13, respectively; Spearman's rank correlation *P* values of 7.04 × 10^−7^, 0.01 and 0.009, respectively). The correlations were still weak when we plotted the codon conservation rates for each individual set of synonymous codons (Supplementary Figure S1). The most conserved codons for Pro and Ala are each the ‘best’ codon in all three metrics, but the most conserved codons for Ile (AUA) and Leu (CUU) have low rankings in all three metrics. Moreover, the Arg CGA codon, the lowest ranking Arg codon in all three metrics, is more conserved than three other Arg codons. Thus, codon conservation is unequal and is also not solely determined by retention of codons with optimal translation efficiency.

We evaluated codon pair conservation rates to uncover the relationship between codon conservation and codon pair conservation. Since codon pairs can affect translation in a manner distinct from their constituent codons, we expected selective pressure on some pairs might differ from that on their constituent codons. On the other hand, if codon pair conservation rate is primarily determined by effects of individual codons on translation or by neutral processes (such as GC content), then the codon pair conservation rate should be directly related to the codon conservation rates of its two constituent codons. To evaluate this model, we examined the fit of a line comparing codon pair conservation rate to the product of the codon conservation rates of its constituent codons (Figure 1B; Supplementary Figure S2) and observed a line (slope = 1.21) with an *r*^2^ value of 0.903, validating that most codon pairs are not evolving independently of their constituent codons.

We found that nine of the 17 previously identified ICPs (25) were among the 40 codon pairs that are more highly conserved as codon pairs than predicted based on the conservation of their constituent codons. Codon pairs that are more than three standard deviations (σ) from the best-fit line were considered to exhibit significantly less or greater codon pair conservation than that predicted by their constituent codons. Only 13 codon pair outliers were >3σ below the line, 12 of which include the single codon amino acids Met AUG and/or Trp UGG. Forty codon pair outliers (including 9 ICPs) were >3σ above the line, revealing greater conservation of these codon pairs than predicted from their constituent codons. Indeed, seven of the nine highly conserved ICPs were more than 6σ from the line, and the three codon pairs with the highest deviation from the best fit line, CGA-CGA (17.8σ), CGA-CCG (10.6σ), and CUC-CCG (8.4σ), correspond to three of four ICPs with highest inhibitory effect on *in vivo* expression (syn-GFP^SEQ^ median score 0.44) (Figure 1C, Supplementary Table S1). We note that highly conserved outliers are not enriched for either the universally avoided or preferred codon pairs (Supplementary Figure S3A) (61), but highly conserved outliers are enriched for low occurring codon pairs. Ten of these outliers (four of which are ICPs) are among the 40 least occurring codon pairs in the yeast ORFeome (Supplementary Figure S3B). However, as we discuss below, low occurrence is neither necessary nor sufficient for conservation.

To determine if the high conservation of ICPs is due to the codon composition of these pairs, we chose a set of corresponding alternative codon pair (ACP) controls. To maintain codon and amino acid composition, the reverse codon pair of each ICP was selected to be the ACP for that pair. In the case of five ICPs, reversal of codons resulted in a codon pair that was also an ICP. In these cases, a different ACP was selected using codons with similar CAI values (Supplementary Table S2). Only one alternative codon pair, CGC-CGA, was an outlier at >3σ above the line (3.36σ) (Supplementary Table S1).

The ICPs were significantly enriched among the conserved codon pairs: 53% (9) of the 17 ICPs were highly conserved codon pair outliers, while only 0.86% (31) of the remaining 3,704 codon pairs and 5.9% (1) of the 17 ACP controls were highly conserved codon pair outliers. Thus the likelihood that 9 of the 17 ICPs would be present by chance among the 40 outliers was 1.7 × 10^−14^, according to the Fisher's exact test. We considered that conservation of the nine ICPs was evidence of selection of these pairs, because the ICPs were identified in an independent assay based on their effect on expression, an assay completely unrelated to conservation.

### High conservation of ICPs is found in multiple pairwise comparisons of species

**Figure 2.**
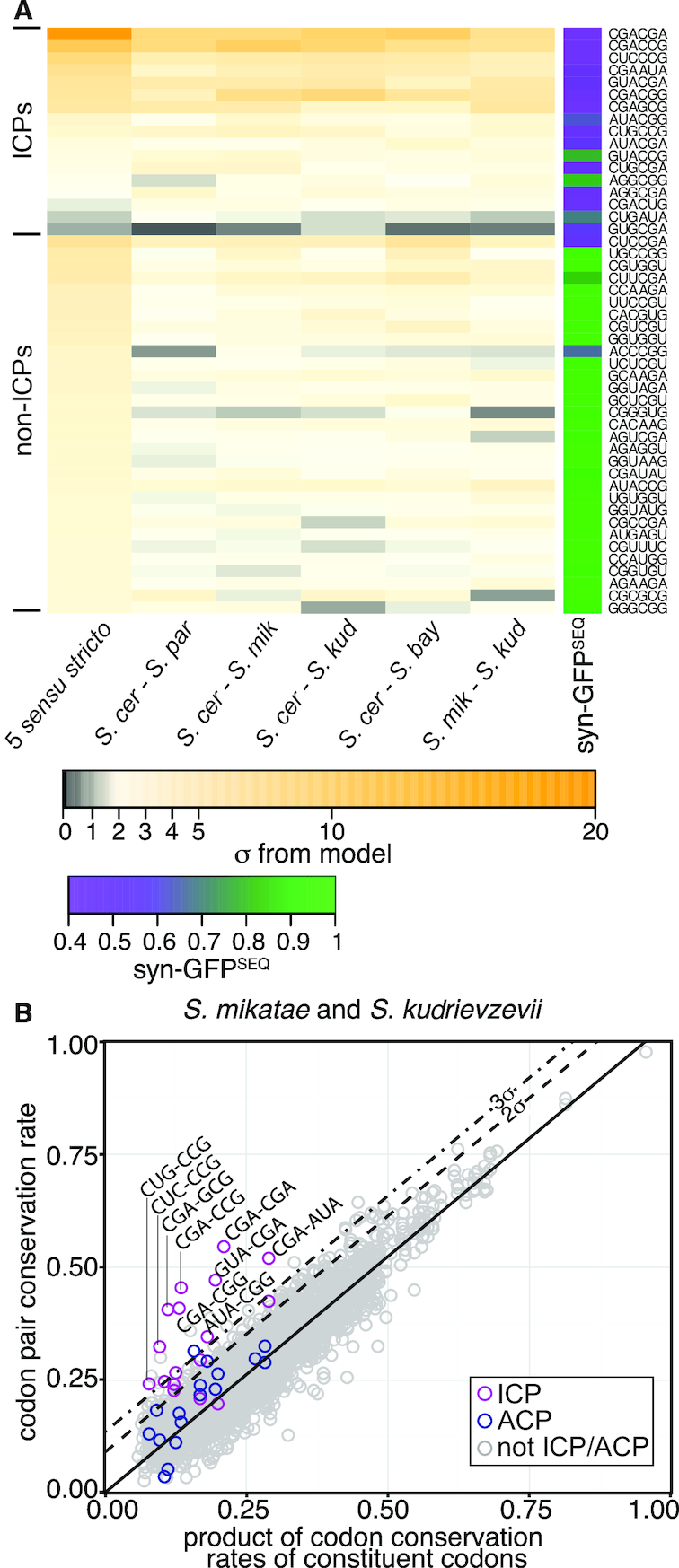
Conservation of ICP_cons_ is apparent across multiple pairwise comparisons within *Saccharomyces sensu stricto* and correlates with inhibitory effects. (**A**) Heat map representation of σ values of 17 ICPs as well as non-ICP outlier codon pairs from the five-species alignments and the corresponding σ values from the indicated pairwise alignments within the *Saccharomyces sensu* stricto yeasts. Syn-GFP^SEQ^ (from 25) of the relevant codon pairs is also shown. (**B**) Scatterplot of codon pair conservation in relation to the product of the conservation rates of its constituent codons across the pairwise alignments of *S. kudriavzevii* and *S. mikatae*.

To determine if the high conservation of these nine ICPs within the *Saccharomyces sensu stricto* clade is due to high conservation among most individual species, or is dominated by one comparison, we examined codon conservation rates and codon pair conservation rates in pairwise species alignments. We compared *S. cerevisiae* orthologs to each of four other *Saccharomyces sensu stricto* species (*S. paradoxus*, S. *mikatae, S. kudriavzevii*, and *S. bayanus* var. *uvarum*) (Supplementary Tables S3–6) and compared orthologs of S. *mikatae* to *S. kudriavzevii* (Supplementary Table S7). Each pairwise comparison detected a range of 8–11 ICP outliers, and 15–20 additional non-ICP outliers that were >3σ from the best-fit line (Figure 2; Supplementary Figure S4A-D), and thus considered, as described above, to exhibit significantly greater codon pair conservation than that predicted by their codons.

The high conservation of the nine ICPs observed in the five species alignment was also observed in the pairwise comparisons (Figure 2A). Seven of these ICPs (CGA-AUA, CGA-CCG, CGA-CGA, CGA-CGG, CGA-GCG, CUC-CCG, and GUA-CGA) were detected as highly conserved outliers (>3σ from the line in all five pairwise comparisons), and all nine of these ICP_cons_ pairs were >2σ from the line in all five comparisons (Supplementary Figure S4A-D). Moreover, all nine of these ICPs were detected as outliers at >3σ in the pairwise comparison between *S. mikatae* and *S. kudriavzevii*, indicating that the conservation of these nine ICPs did not reflect an *S. cerevisiae*-specific phenomenon (Figure 2B).

Several of the eight ICPs that were not outliers in the five species alignment were also conserved based on the pairwise comparisons. Five of these ICPs were >2σ from the best fit line in four or five comparisons (Figure 2A). Thus, a mark of codon pair conservation extended to 14 of the 17 ICPs.

Most non-ICP codon pair outliers were not consistently highly conserved across the five pairwise comparisons (>3σ). Only three of the 31 non-ICPs were highly conserved across all the pairwise alignments (>3σ from the line), and only 8 of these were >2σ from the line in all pairwise comparisons (Figure 2A). Based on the criterion of consistent conservation of outliers from the five species comparison (>2σ in all pairwise comparisons), 53% (9) of the 17 ICPs were highly conserved, while only 0.2% (8) of all other (3704) codon pairs are highly conserved. Thus, we continued to investigate the basis for conservation of these nine ICPS, and called this set of nine inhibitory codon pairs, the ICP_cons_ set.

### High conservation of the nine ICP_cons_ is not due to the encoded dipeptide, sequence motifs or their location in highly conserved sequences, but is related to their position within the coding sequence

We considered the possibility that these ICP_cons_ are highly conserved because they encode highly conserved dipeptides, rather than highly conserved codon pairs. To compare conservation rates among codon pairs that specify the same dipeptide, we calculated a normalized conservation score for each codon pair:

(3) \begin{eqnarray*}&&Normalized\ conservation\ score\ =\nonumber\\ && \frac{{codon\ pair\ conservation\ rate}}{{codon1\ consv\ rate\ \times \ codon2\ consv\ rate}}\end{eqnarray*}

The normalized conservation scores vary substantially even within a single dipeptide family: the Ile-Arg dipeptide family ranges in normalized conservation scores from 0.62 to 4.2 while the Arg-Ala dipeptide family ranges from 0.43 to 16. The normalized conservation score showed that the nine ICP_cons_ are more highly conserved than any other means of encoding their respective dipeptides, with seven ICP_cons_ ranking first and two ICP_cons_ ranking second because the highest ranking conserved synonymous pair is an ICP_cons_ (Supplementary Table S8).

**Figure 3.**
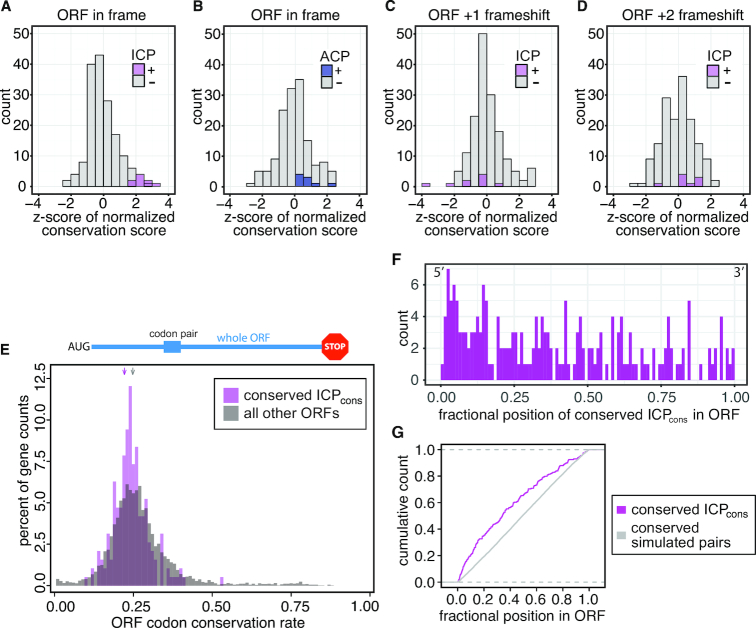
High conservation of the nine ICP_cons_ is not due to the encoded dipeptide, sequence motifs, or their location in highly conserved sequences, but is related to their position within the coding sequence. (A–D) ICP_cons_ are more highly conserved than synonymous codons encoding the same dipeptide. (**A**) A histogram of the *z*-scores based on the normalized conservation scores of the ICP_cons_ and their synonymous codon pairs. A total of 168 codon pairs encode the seven dipeptides specified by the nine ICP_cons_; the y axis, labeled count, indicates the number of these codon pairs with the indicated *z*-score. (**B**) Histogram of the *z*-scores for alternative codon pairs (ACPs). (**C**, **D**) Histograms of the *z*-scores for the ICP_cons_ sequences in the +1 and +2 reading frame. (**E**) Conservation of ICP_cons_ is not due to their location in highly conserved ORFs. Histograms show the codon conservation rates of ORFs with conserved ICP_cons_ (magenta) and of all other genes (gray). Arrows mark the median codon conservation scores. (**F**) Histogram of the relative positions of conserved ICP_cons_ pairs in ORFs, with positions indicated as a fraction of the length of the ORF. (**G**) Cumulative distribution of relative position of conserved occurrences of ICP_cons_ (magenta) compared to conserved occurrences of codon pairs in 100 simulations, each with nine randomly chosen codon pairs (gray).

We compared conservation rates across dipeptides, calculating a z-score for each codon pair (the number of σ from the mean normalized conservation score within the dipeptide family):

(4) \begin{equation*}z\ = \ \frac{{X_{\rm cp}\ - \ \mu_{\rm dp}}}{{\sigma_{\rm dp}}}\end{equation*}

where *X*_cp_ is the normalized conservation score, *μ*_dp_ is the mean normalized codon conservation score for the particular dipeptide family, and *σ*_dp_ is the σ for the dipeptide family. The *z*-scores for the nine ICP_cons_ are clustered at the right tail of the distribution of the *z*-scores of synonymous codon pairs (Figure 3A), suggesting that the high conservation of the ICP_cons_ is still observed within the context of their dipeptide families. The analogous z-scores for the ACPs were also higher than the z-scores for their synonymous codon pairs (Figure 3B), but not to the same extent as the ICPs. We also showed that the *z*-score effectively discriminated ICP_cons_ from synonymous pairs but did not do so for ACPs using receiver-operator characteristic curves (Supplementary Figure S5A and B).

We considered the possibility that the ICP_cons_ were highly conserved as an artifact of highly conserved RNA motifs. If the conservation is related to the nucleotide sequence, then we would observe similar conservation for the sequence regardless of the reading frame. Thus, we calculated the *z*-score of the normalized codon pair conservation score for each of the nine ICP_cons_ out of phase with the reading frame, by shifting the reading frames by +1 or +2 nucleotides (Supplementary Table S9). High relative conservation of the ICP_cons_ six-nucleotide sequences is not observed in either the +1 or +2 frames (Figure 3C, D; Supplementary Figure S5C, D, Supplementary Table S9). Thus, conservation of these ICP_cons_ pairs is not due to conserved sequence motifs, but, rather, is likely due to a function that is linked to translation.

We considered the possibility that these codon pairs appeared to be highly conserved because their constituent individual codons are among the least selected codons in the yeast genome. Seven codons (CGA, CGG, AUA, CCG, CUC, GCG, GUA) that account for 17 of the 18 codons found in these nine highly conserved pairs have seven of the eight lowest selection coefficients (modeled based on gene expression levels in *S. cerevisiae*) (15). Therefore, we examined the codon pair conservation rate (Equation 2) among the family of dipeptides for each codon pair without normalization for constituent codons. Remarkably, seven of the nine ICP_cons_ are more highly conserved than any other means of encoding their respective dipeptides (except another ICP_cons_), even without controlling for their constituent codons (Supplementary Table S8).

We also demonstrated that conservation of these nine ICP_cons_ is not due to the conservation of either the genes or locations in which they are found. To obtain a score for conservation of an entire gene, we calculated an ORF codon conservation rate as the fraction of all codons in that ORF that are conserved:

(5) \begin{eqnarray*}&&ORF\ codon\ conservation\ rate \nonumber\\ &&= \frac{{conserved\ codon\ counts\ in\ ORF}}{{reference\ ORF\ length\ in\ codons}}\ \end{eqnarray*}

We compared these ORF codon conservation rates between the set of ORFs containing conserved occurrences of ICP_cons_ and the set of all other ORFs. Median ORF codon conservation rates for *S. cerevisiae* ORFs with conserved ICP_cons_ were lower than the corresponding median values for all other *S. cerevisiae* ORFs (0.24 compared to 0.25) (Figure 3E). The ORF conservation rates of ORFs with the ICP_cons_ was also similar to that of a subset of 2,889 ORFs with comparable codon usage to ORFs with the ICP_cons_ (0.23). Thus, the ORFs that contain conserved instances of the ICP_cons_ are not themselves generally more conserved than average. Additionally, no individual ICP_cons_ is associated with ORFs that are highly conserved (Supplementary Figure S6A). Furthermore, the conservation of the ICP_cons_ is not explained by placement within local clusters of high conservation (Supplementary Figure S5E). These results suggest that conservation of the nine ICP_cons_ is not due to a selective location in highly conserved *S. cerevisiae* ORFs.

We did find evidence that the position of conserved occurrences of ICP_cons_ within genes is not random, but biased towards the 5′ end of the gene. To this end, we examined the relative position of conserved ICP_cons_ pairs as well 100 simulations of nine randomly chosen codon pairs. Nearly half of the conserved occurrences of ICP_cons_ pairs are found within the first third of the gene (median of 0.34), while the position of the simulated pairs maps to the expected average median of 0.51±0.01 (Figure 3F and G). Three additional observations highlight the importance of the location of the conserved ICP_cons_ pairs. First, the positional bias towards the 5′ end is at least partially related to conservation, because the complete set of ICP_cons_ pairs in the *S. cerevisiae* genome exhibit a more random distribution (median position of 0.42) (Supplementary Figure S6B, C). Second, the position of the ICP_cons_, rather than gene identity, is a major factor in its conservation; 75% of genes that contain an ICP_cons_ in all five *sensu stricto* species (258 genes) contain the same ICP_cons_ in the same position in all five species (194 genes). Third, position of the ICP_cons_ pair is likely important, because the conserved pairs overlap in three of the six genes with multiple occurrences of conserved ICP_cons_ pairs.

### Slow translation rates are correlated with extreme conservation of codon pairs

To begin to understand the possible function(s) of the highly conserved pairs, we examined the properties of ICP_cons_ and of the genes that contain them. We previously used existing ribosome footprinting data of native *S. cerevisiae* genes to assess the density of ribosome footprints for the 3,721 sense codon pairs across a 100 codon window surrounding the pair. We reported that twelve of the ICPs were among the 24 codon pairs with the highest relative cumulative ribosome occupancy (>3σ above the mean) at the A-P and P-E sites of the ribosome (25). We inferred that these 12 ICPs were translated slowly based on the accumulation of ribosomes at these codon pairs, implying that slow translation at these codon pairs is part of the mechanism for reduced expression.

**Figure 4.**
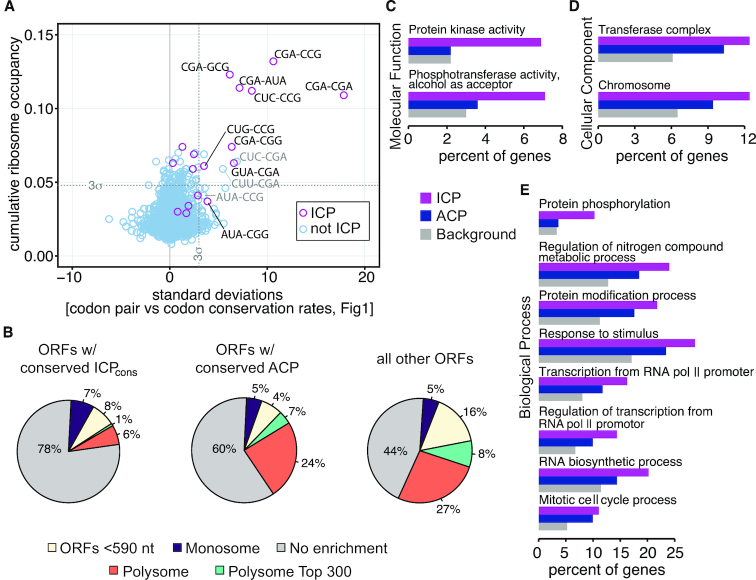
Unique translational properties and specific functional categories of the conserved ICP_cons_. (**A**) ICP_cons_ are slowly translated. Plot of cumulative ribosome occupancy at each codon pair (values from Gamble *et al.* (25)) versus the standard deviation from the best fit line of codon pair conservation rate as a function of the product of the codon conservation rates. (**B**) The distribution of genes across translational categories defined by Heyer and Moore (60). Genes in which ICP_cons_ are conserved are compared to genes in which the ACPs are conserved and to all other genes in these alignments. Chi-squared test had a *P*-value <2.2 × 10^−16^ (**C**–**E**) Gene ontology enrichment analysis of ICP_cons_-conserved ORFs and ACP-conserved ORFs, compared to all yeast ORFs (labeled background). Only categories in which *P* values for ICP_cons_ conserved-ORFs are ≤0.01 are shown; individual *P* values and gene identity are reported in Supplementary Tables S13–S17.

In examining the ICP_cons_, we found a strong correlation between the degrees of conservation of the ICP_cons_ and ribosome occupancy. To demonstrate this, we plotted the cumulative ribosome occupancy as a function of the deviation from the best fit line for codon pair conservation rate versus codon conservation rate product (Figure 4A; Supplementary Table S10). This best fit line is shown in Figure 1B, and the deviation is the number of σ from the line. The four most highly conserved codon pairs in our analysis (ranging from 7.15 to 17.85σ above the mean) were among the five codon pairs in *S. cerevisiae* with the highest relative ribosome occupancy, and the eight most highly conserved codon pairs (with >6σ above the mean) were among the top 18 codon pairs in ribosome occupancy. Moreover, seven of these eight codon pairs are known ICPs, all of which were outliers (> 3σ) in every *Saccharomyces sensu stricto* pairwise comparison. The eighth pair (CUC-CGA, not an ICP) was identified as an outlier in all individual comparisons, and had a low synGFP^SEQ^ score (0.56) (25), but was not identified as an inhibitory pair by enrichment of its sequence in low expression variants, likely due to under-representation of the sequence in our library. The two remaining conserved ICP_cons_ rank 19th (CUG-CCG) and 202nd (AUA-CGG) in ribosome density, while the other two non-ICP pairs conserved in all five individual comparisons (CUU-CGA and AUA-CCG) rank 23rd and 134th in ribosome density. Based on these results, we infer that conservation is related to an accumulation of ribosomes at these pairs, presumably indicating a local slowing of translation.

### Genes with conserved ICP_cons_ have unique translational properties in *S. cerevisiae*

To discern likely functions of ICPs, we looked for properties that distinguish ORFs in which the nine ICP_cons_ are conserved from other ORFs. We examined the characteristics of the set of 364 genes in the *Saccharomyces sensu stricto* species in which a particular ICP_cons_ was present at the same location in four or five species (Supplementary Table S11). We compared the characteristics of this set (called ICP_cons_-conserved ORFs), to characteristics of two other sets of ORFs: the corresponding ACP-conserved ORFs (223 ORFs) (Supplementary Table S12) and all other ORFS in our analysis dataset (3459 ORFs). These groups of ORFs were compared across several metrics to determine unique characteristics of the ICP_cons_-conserved ORFs. In particular, the comparison between the ICP_cons_-conserved ORFs and the ACP-conserved ORFs was used to distinguish properties that were specifically associated with the ICP_cons_-conserved ORFs from those that might generally be associated with genes encoded with suboptimal codons.

The ICP_cons_-conserved ORFs and the ACP-conserved ORFs are relatively well-matched sets of ORFs in that both encode proteins with similar mean and median CAI values and length (Table 1). The increased mean and median lengths of these ORF sets (as well as their distributions, Supplementary Figure S7A) distinguish these groups from the set of all other ORFs. The increased lengths are likely because both ICP_cons_ and ACPs are composed of rare codons. As reported previously (33), longer genes have a higher probability of containing rare codons by chance alone. We find no enrichment of ICP_cons_ per unit of length in long genes compared to other genes.

We considered that the translational properties of ORFs with the ICP_cons_ might differ from the translational properties of most other genes because the ICPs were identified based on their inhibitory effects on expression and were composed of the most slowly translated codon pairs in yeast. Therefore, we investigated the relative density of ribosomes on ORFs from the ICP_cons_, ACP and other gene sets, using the evaluation of gene distribution on monosomes and polysomes by Heyer and Moore (60). In this analysis, ORFs were categorized into five groups based on an estimate of the average number of ribosomes translating the ORF and on the size of the ORF: (i) ORFs shorter than 590 nucleotides, which were mostly on monosomes, (ii) monosomes (for ORFs >590 nucleotides), (iii) no enrichment (ORFs that do not meet the cutoff for either monosome or polysome enrichment), (iv) polysomes and (v) polysome top 300 ORFs with the highest number of ribosomes (60).

We found a striking specific depletion of the ICP_cons_-conserved ORFs from the polysome set relative to the distributions from ACP-conserved ORFs and all other ORFs (Figure 4B). There is a unique depletion in polysome association among the ORFs in the ICP_cons_ set; <7% of these ORF are associated with polysomes (both polysomes and top 300 categories) compared to 31% in the ACP set and 35% in the ‘all other ORFs’ set. Furthermore, failure of ICP_cons_-conserved ORFs to associate with polysomes is not due to small size, since as expected both the ICP_cons_-conserved ORFs and the ACP-conserved ORFs are depleted in ORFs <590 nucleotides, consistent with the increase in ORF length in both of these sets (Table 1). The depletion of polysomes from ICP_cons_ mRNAs is unexpected in the context of the high ribosome density at the ICP_cons_ pairs themselves, but might reflect effects of ribosome collisions at these ICPs (62) or an interplay between elongation and initiation, as proposed by others (63,64) and discussed more completely below in the Discussion. Essentially, polysome depletion from this set strongly indicates an impact of ICP_cons_ on translation, but the means by which this occurs is unknown.

The depletion of polysomes on the ICP_cons_ set of ORFs would be expected to reduce protein production. Indeed, we find that the ICP_cons_ set of ORFs differ substantially from both the ACP and all other ORFs sets in the reduced abundance of proteins encoded by the ICP_cons_ set. The mean copy number of ICP_cons_ proteins is 435 molecules per cell based on mass spectrometry measurements (58), but is 9,748 for the ACP set and 10,170 for all other genes (Table 1). Similar results are obtained from analysis of Tap-tagged genes by Western (57) (Table 1). Similarly, it has been established that mRNA half-lives are generally correlated with codon usage (65) and that even faster mRNA decay is associated with ICPs (66); we found that the mean, and median mRNA half-life of genes with ICP_cons_ were reduced, compared to that of the other gene sets (Table 1). The distribution of mRNA half-lives for each set of ORFs is shown in Supplementary Figure S7B.

The ICP_cons_ conserved genes are specifically enriched for genes that encode protein kinases and genes involved in protein phosphorylation (Figure 4C–E; Supplementary Tables S13–S17), based on Gene Ontology enrichment analysis (67). The significant enrichment categories (*P* value ≤0.01) are greater for the ICP_cons_ gene set than for the ACP (Supplementary Tables S14 and S17), but this may be due to the larger number of genes in the ICP_cons_ set (364 versus 223), which provides a greater power for finding significant categories for the ICP_cons_ set. Therefore, in each category in which significant enrichment was reported for the ICP_cons_ genes, we directly compared the percentage of ICP_cons_ and ACP genes that were members of that category. If the ICP_cons_ genes are actually enriched with respect to a particular function, we expected a greater percentage of the 364 ICP_cons_ genes in the GO category compared to the percentage of the 223 ACP genes. Nearly 7% of the genes in the ICP_cons_ set are annotated with a molecular function of ‘protein kinase activity’ while only 2.2% of both the ACP and total gene sets are annotated in this manner (Figure 4C). In most other categories, from mitotic cell cycle to regulation of transcription from pol II promoter and response to stimulus, we noted similar enrichments in both the ICP_cons_ and ACP sets. The enrichment of ICP_cons_ and ACP sets in similar functions may point to particular gene sets in which suboptimal codon use is important for function and is thus conserved.

### Highly conserved codon pairs are found in *Candida* species and contain codons decoded by I•A and U•G wobble interactions

**Figure 5.**
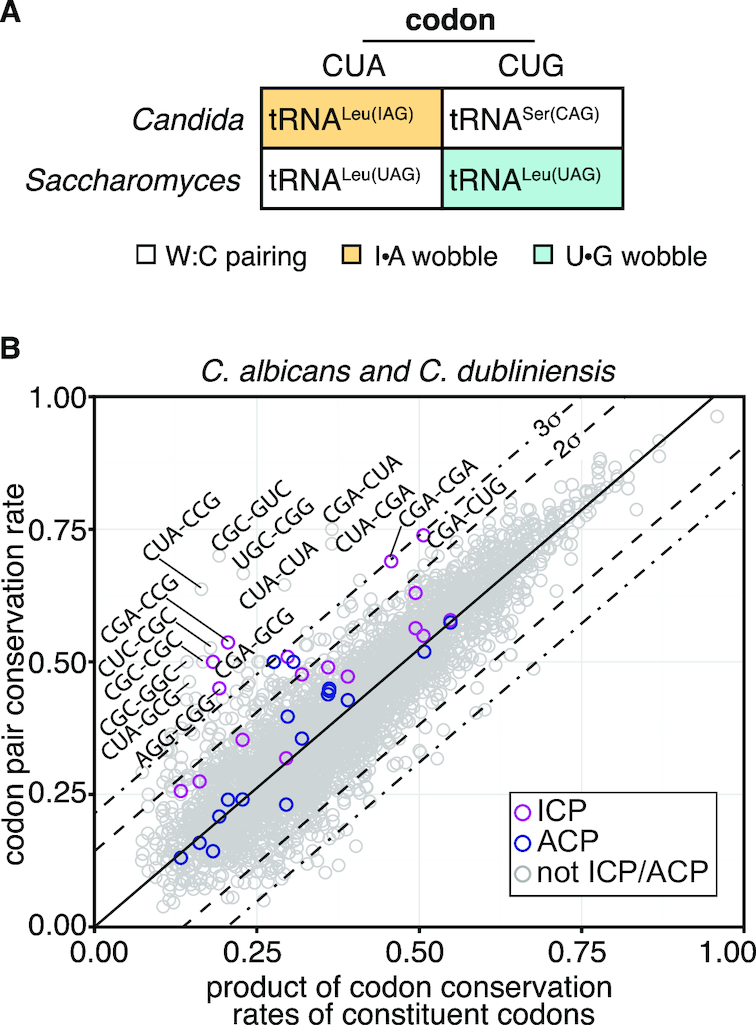
A comparison of codon pair conservation between *Candida albicans* and *Candida dubliniensis* yields outlier pairs composed of codons decoded by I•A wobble interactions. (**A**) Schematic of differences between *C. albicans* and *S. cerevisiae* in decoding CUA and CUG codons. (**B**) Scatterplot of codon pair conservation in relation to the product of the conservation of its constituent codons across the pairwise alignments of *C. albicans* and *C. dubliniensis* ORFs.

To determine if enhanced conservation of particular codon pairs is observed beyond the *Saccharomyces sensu stricto* species, we examined the conservation rates of codons and codon pairs between two species in the *Candida* clade, *C. albicans* and *C. dubliniensis* (Supplementary Table S18). *C. albicans*, although still a member of the hemiascomycetes class, diverged from *S. cerevisiae* ∼270 million years ago (68–70). *C. albicans* retains many aspects of *S. cerevisiae* decoding, but differs in two key aspects (Figure 5A). First, in *S. cerevisiae*, the CUA codon is decoded as Leu using an exact match (no wobble interaction) tRNA, but in *C. albicans*, the CUA codon is decoded using an I•A wobble interaction. Second, in *S. cerevisiae*, the CUG codon is decoded as Leu using a U•G wobble, but in *C. albicans*, the CUG codon is decoded as Ser using an exact match tRNA (69).Thus, if enhanced conservation of codon pairs is in part due to wobble decoding, we expected that the CUA codon would be enriched in conserved codon pairs in *Candida* species.

Conservation of codon pairs in *Candida* is generally directly related to the conservation of the constituent codons; the best fit line (slope 1.03) had similar fit with an *r*^2^ of 0.97 (Figure 5B). However, similar to our observations with the *Saccharomyces* species, there were 32 codon pairs that were more highly conserved than expected (outliers >3σ above the line) and 11 codon pairs that were less conserved than expected (>3σ below the line).

The highly conserved codon pairs in *Candida* are enriched for codons decoded using I•A and U•G wobble interactions (Figure 5B), substantiating the idea that conservation of particular codon pairs is due to their inefficient decoding properties. Both codons decoded by an I•A wobble interaction (CUA, CGA) in *Candida* are found in the highly conserved pairs. The CUA codon is found in five of the 32 conserved codon pairs (CUA-CCG, CUA-CUA, CUA-CGA, CGA-CUA, CUA-GCG) in the *Candida* species comparison, although it was not found in any conserved pair in the *Saccharomyces sensu stricto* species. Of note, for three CUA-containing pairs in *Candida*, there is a corresponding *S. cerevisiae* ICP, specified with the CUG codon (CUG-CCG, CUG-CGA, and CGA-CUG). The CGA codon is found in four pairs in the *Candida* comparison, and was found in ten of 40 highly conserved codon pairs in *S. cerevisiae*. In addition, two codons decoded by U•G wobble in *Candida* (Ala GCG and Pro CCG) are found multiple times in the *Candida* conserved codon pairs, occurring nine and three times respectively (compared to two and four occurrences in *Saccharomyces sensu stricto* species). Based on the multiple occurrences of CGA, CUA, GCG, and CCG codons in these highly conserved pairs, we speculate that some of the highly conserved codon pairs in the *Candida* species likely have similar slow translation, as seen with the nine ICP_cons_ pairs in the *Saccharomyces sensu stricto* species. Conservation of these pairs thus implies the importance of a similar method of translational regulation in these distant species.

## DISCUSSION

We demonstrate evolutionary selection for a set of inefficiently translated codon pairs at defined locations in yeast; these inhibitory codon pairs (ICPs) also mediate slow translation. Thus, we infer that these ICPs have a biological function, most likely to slow translation. Nine of 17 previously identified ICPs, composed solely of suboptimal codons, are extremely conserved relative to the conservation of their constituent codons across the coding regions of five *Saccharomyces* *sensu stricto* yeast species. The set of 17 ICPs, which were identified based on their significant reduction of *in vivo* expression relative to their synonymous optimal codons, exert their effects in a manner distinct from those of their constituent codons and many are slowly translated (25). Thus, their identification was independent of conservation. Conservation of the nine ICPs is observed across pairwise alignments, is enriched relative to conservation of all other codon pairs (53% versus 0.8%), is not due primarily to dipeptide identity, sequence motifs or the conservation of genes or locations in which they occur. Seven of these nine ICPs are the most highly conserved codon pairs coding for their respective dipeptides, without regard for conservation of their constituent codons. As we observed previously in identification of the ICPs (25), the order of the codons is also important for their conservation and ICP function; among the Arg-Arg pairs, CGA-CGG is an ICP and highly conserved (3/18), while CGG-CGA is not an ICP and is not highly conserved (0/17). Moreover, codon pair conservation is strongly correlated with high ribosome occupancy at the pair, i.e., those ICPs with the highest ribosome density are most conserved as codon pairs, suggesting a selection for slow translation.

We suggest that this method of regulating translation has been preserved across diverse Ascomycetes fungi. The high conservation of a specific subset codon pairs, composed entirely of suboptimal codons, is not unique to the *Saccharomyces sensu stricto* yeasts. Pairwise alignments in *C. albicans* and *C. dubliniensis*, which diverged from *S. cerevisiae* ∼270 million years ago, showed strong conservation of 32 codon pairs. Based on these results, we infer that selective pressure is exerted on particular codon pairs across the Ascomycetes fungi to maintain slow translation at defined locations. Moreover, a limited set of dipeptides may be suited to this regulation, since 15 dipeptides are found among the conserved codon pairs in both sets of species (with fewer than 30 total dipeptides specified by the outliers in either set).

The identity of codons in conserved pairs in both *Saccharomyces* and *Candida* species reinforces the importance of wobble decoding, in particular I•A wobble decoding. The CGA codon, the only codon decoded by I•A wobble in *S. cerevisiae*, occurs 10 times in the 40 highly conserved codon pairs in the *Saccharomyces sensu stricto* species, and four times in the 32 highly conserved codon pairs in the *Candida* species. Likewise, the CUA codon, decoded by I•A wobble in *C. albicans* and *C. dubliensis*, occurs five times in the highly conserved pairs in the *Candida* species. The importance of I•A wobble decoding is underscored by retention of this inefficient decoding strategy throughout the hemiascomycetes fungi, despite the fact any of 17 single point mutations in *S. cerevisiae* could eliminate it (71–73). Thus, we infer that species-specific, inefficient decoding strategies are key to the identity of highly conserved codon pairs.

The substantial effects of the ICP_cons_ pairs on translation and gene expression are underscored by three observations. First, expression of genes in which ICP_cons_ are conserved is markedly reduced. Protein levels from genes in which the ICP_cons_ are conserved are estimated to be between 5% - 31% of those from all other genes, including genes in which ACPs are conserved. Second, the ICP_cons_ include the six codon pairs with the highest ribosome occupancy in native yeast genes. This correlation between conservation of codon pairs and ribosome occupancy points to selection for slow translation in the *Saccharomyces sensu stricto* yeasts. Third, the genes in which ICP_cons_ are conserved have a reduction in ribosomes per mRNA. Based on ribosome profiling data distinguishing monosomes from polysomes (60), only 7% of the mRNAs with ICP_cons_ are found in polysomes, despite their generally longer length than all other ORFs. Thus, despite the high density of ribosomes at the ICP_cons_, the overall density of ribosomes on these mRNAs is substantially reduced. The depletion of ribosomes from these mRNAs might be explained by the observation that mRNA decay and aborted translation can result from collisions between stalled ribosomes (62). Thus, genes with ICPs might undergo these collisions, reducing their apparent ribosome content, or might be engineered to avoid such collisions, by reducing their initiation rates. Others have demonstrated that there is an interplay between translation elongation and initiation, in particular demonstrating that the effects on translation output caused by elements that slow elongation are strongly influenced by 5′ elements that control initiation (63,64). Such mechanisms might also explain some of the effects of the ICP_cons_ on translation.

The function(s) of the ICP_cons_ are unknown, but we speculate on three possible functions that derive from their known effects on translation or expression. First, the primary function of the ICP_cons_ pairs might be to bring about translational pausing, which has previously been implicated in regulation of temporal folding of protein structural domains. Indeed, protein secondary structure motifs have been found to have distinct patterns of enrichment of suboptimal or optimal codons (35,39,40,74). Translational pausing might also facilitate co-translational protein interactions; indeed for a number of complexes, such as Trm8 and Trm82 (75), complex formation depends upon co-expression. Second, the ICP_cons_ pairs may primarily function to restrict expression of low expression genes, consistent with the finding that ICP_cons_ are in genes that are primarily low expression. There are a set of proteins, e.g. some cell cycle proteins and transcription factors, whose presence is essential, but in excess these proteins are likely to be deleterious (31,76). Thus it is essential to maintain sufficient mRNA, but to keep expression low. Third, the ICP_cons_ might work to reduce ribosome occupancy of a gene and eliminate ribosome collisions. Recent evidence for ribosome collisions includes the isolation of disomic footprints (77) as well as evidence that such collisions induce the No-Go decay pathway (62).

It is unknown why the selective pressure to retain an ICP_cons_ is apparently greater than the selective pressure to retain a pair of optimal codon pairs. In seven instances, the ICP is the most highly conserved means of encoding a dipeptide, even without normalization for the conservation of its constituent codons. One plausible explanation is that some ICP_cons_ are selected against in most locations and that the remaining occurrences of these ICP_cons_ are those at which they are functionally beneficial. Consistent with this explanation, four of the six most conserved pairs are among the 40 least occurring codon pairs in yeast. However, low occurrence is not necessary for conservation of the ICP_cons_ since none of the remaining five ICP_cons_ pairs is among the 100 least occurring pairs in yeast. The correspondence between low occurrence and conservation is also dubious because only one of the other six low occurring conserved pairs was conserved in the individual species comparisons. A second explanation is that the effects of optimal pairs are rarely position dependent. Thus, in highly expressed genes, there may be an overall selection for optimal codons, but this pressure does not preclude insertion of any single suboptimal codon into most positions.

The ICP_cons_ could be used in conditional regulation of expression, particularly in light of the enrichment of genes involved in phosphorylation among the genes with conserved ICPs. Gamble *et al.* illustrated the ability to rescue expression inhibition by ICPs by introducing non-native cognate tRNAs or over-expressing native tRNAs (25). Thus, the inhibitory effect of ICP_cons_ could be modulated by conditional availability or function of tRNAs, a concept that would not be entirely new. Changes in tRNA pools occur and can be a means to control expression rates under specific circumstances (78). Furthermore, this plasticity to meet changing translational needs goes beyond tRNA pools. tRNAs undergo extensive modifications that can affect their translational efficiency and accuracy (79,80). For instance, stress conditions can result in changes in tRNA modifications that reprogram wobble interactions, causing selective translation of specific mRNAs (81–83).

The remarkable conservation of the most slowly translated codon pairs in *S. cerevisiae* points to a previously unappreciated mechanism of translation regulation that is conserved throughout evolution of the hemiascomycetes fungi. Based on the conservation of similar pairs in distinct clades of *Ascomycotes*, it seems likely that these mechanisms will also be found in higher eukaryotes.

## Supplementary Material

Supplementary Data
