# Supplementary material for: Conservation of location of several specific inhibitory codon pairs in the Saccharomyces sensu stricto yeasts reveals translational selection
Source: Nucleic Acids Res. 2018 Dec 21;47(3):1164–77. doi: 10.1093/nar/gky1262 (PMC6379720; doi:10.1093/nar/gky1262)
Supplement: Supplementary Data [file gky1262_supplemental_files.zip › Ghoneim_Supplementary_Info_2018_11_19.pdf]

Supplementary information

Conservation of location of several specific inhibitory codon pairs in the *Saccharomyces sensu stricto yeasts* reveals translational selection

Dalia H. Ghoneim[1,2], Xiaoju Zhang[1,2], Christina E. Brule[1,2],

David H. Mathews[1,2,3 †], Elizabeth J. Grayhack[1,2,3 †]

[1]Department of Biochemistry and Biophysics, School of Medicine and Dentistry, University of Rochester, Rochester, NY 14642, USA
[2]Center for RNA Biology, University of Rochester, Rochester, NY 14642, USA.
[3]Co-senior author

[†]Corresponding authors

Correspondence: elizabeth_grayhack@urmc.rochester.edu

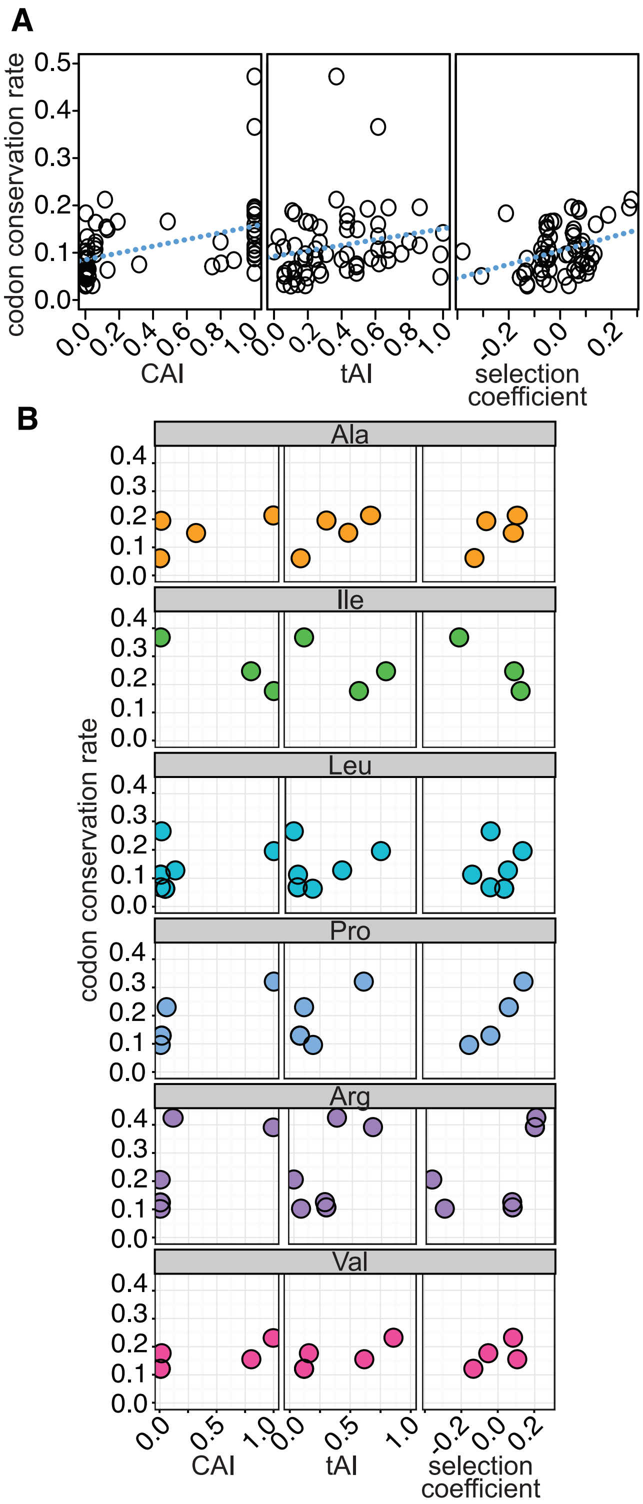

Figure S1. Conservation of codons does not strictly correlate with expression metrics. *(A)* Scatterplots of the codon conservation score in the five species *Saccharomyces sensu stricto* yeast alignments and their corresponding CAI, tAI, and selection coefficients (15) based on expression data. The spearman rank correlation for CAI, tAI, and selection coefficient p-value were 7.03×10^{-7}, 0.010, and 0.009 respectively. *(B)* Scatterplots of codon conservation and expression-based metrics decomposed according to amino acid.

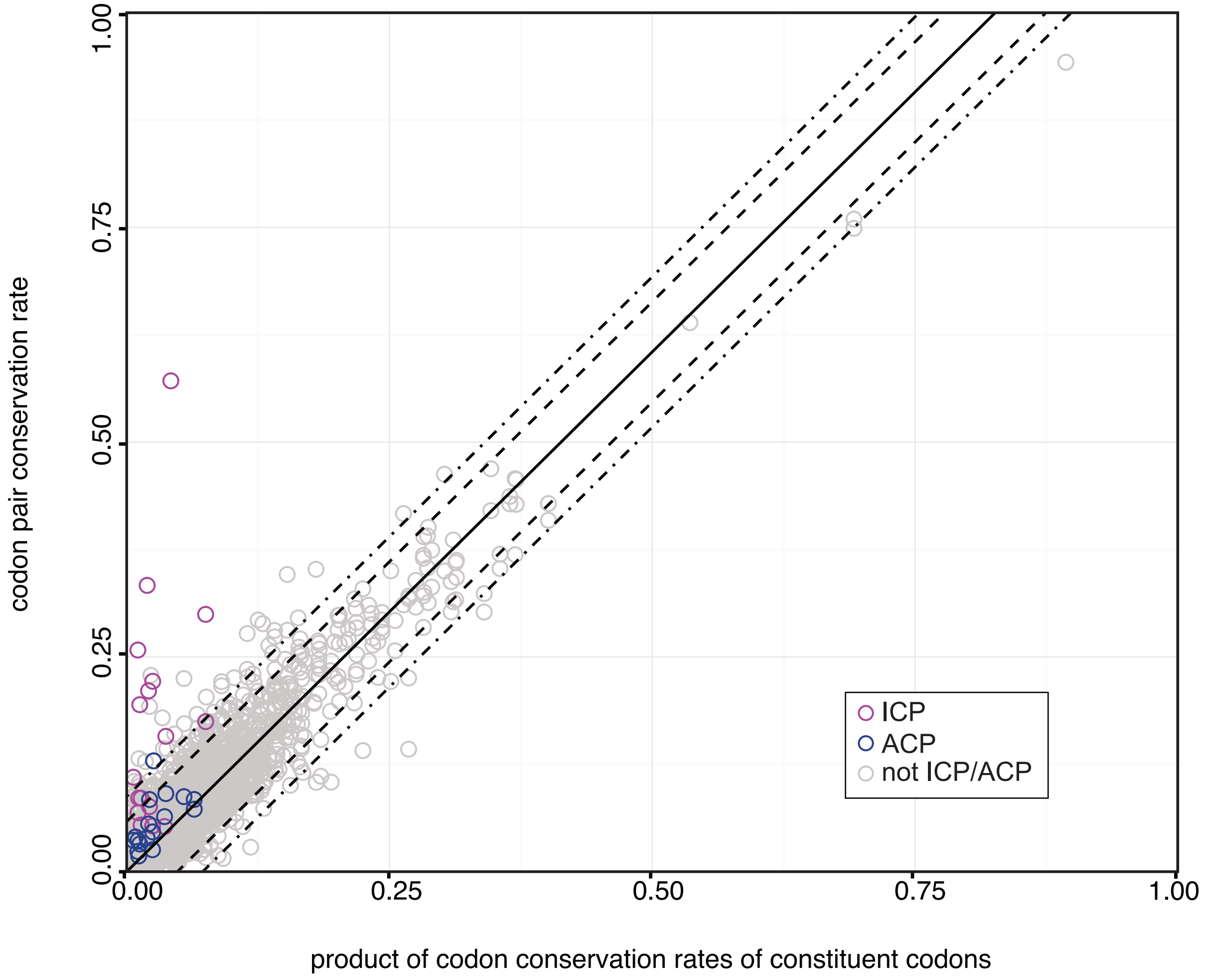

Figure S2 (refers to Figure 1). Full scatterplot of the codon pair conservation rate and the product of the codon conservation rates of the constituent codons. The plot includes 4 codon pairs not shown in Figure 1 (WW, UGG-UGG; WM, UGGAUG; MW, AUGUGG; MM, AUGAUG). The solid line is the best fit line, and dashed lines indicate two and three standard deviations from the best fit line.

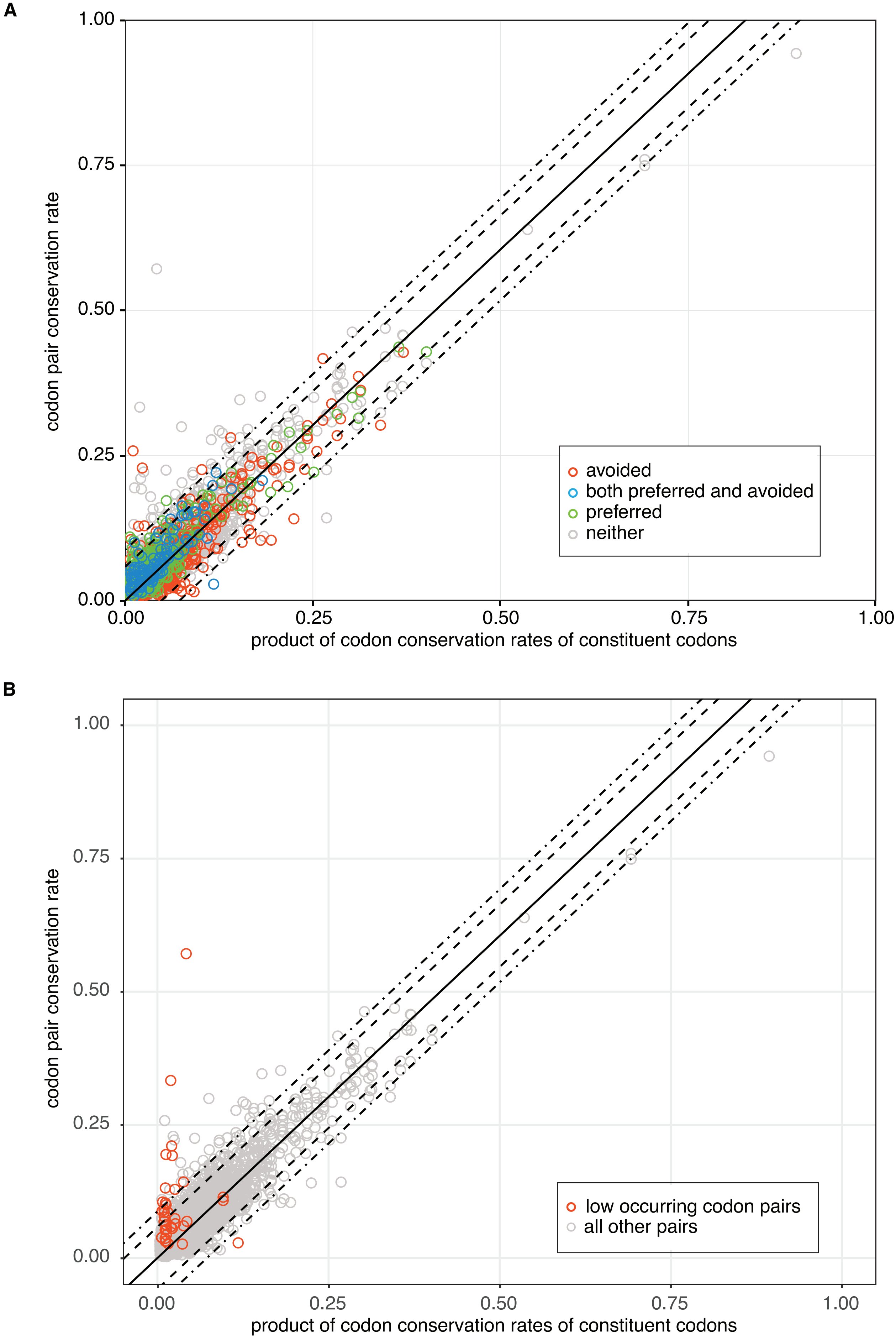

Figure S3. The relationship between conservation and occurrence of codon pairs. *(A)* Codon pairs that are universally preferred (green), avoided (orange-red), or fitting both definitions of preferred and avoided (blue) (as defined in Tats *et al.*) (61) are highlighted in the scatterplot of codon pair conservation rate vs. the product of codon conservation rates. *(B)* The forty least occurring codon pairs (orange-red) in the *S. cerevisiae* genome are highlighted in the same scatterplot.

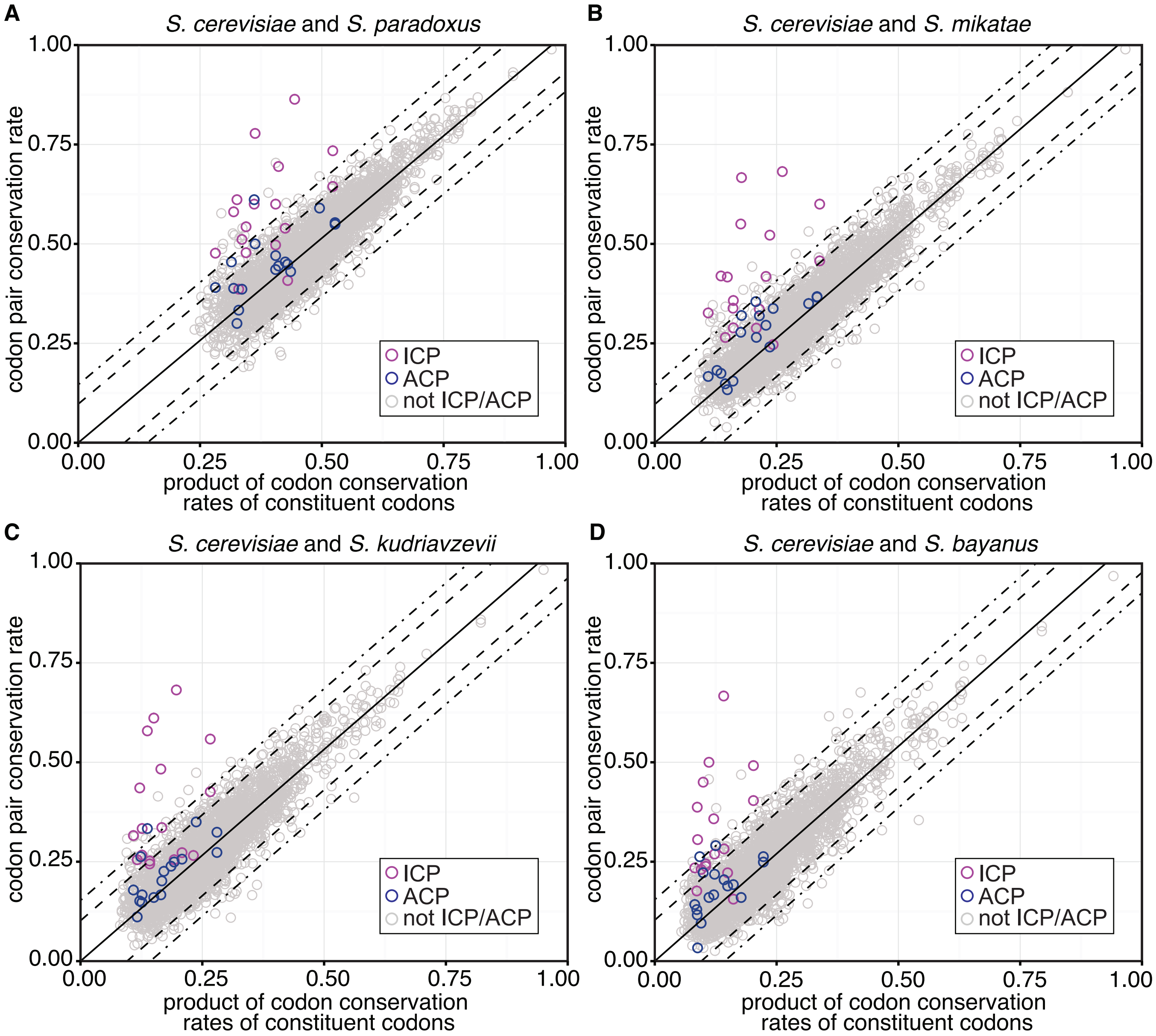

Figure S4 (refers to Figure 2). Pairwise alignments across *Saccharomyces sensu stricto* yeasts consistently yield conserved ICPs. *(A-D)* Pairwise alignments between the indicated species each have eight to 11 ICPs that are highly conserved with σ >3 from the line. The solid line in each graph represents the best fit line and the dashed lines are two and three standard deviations from the best fit line.

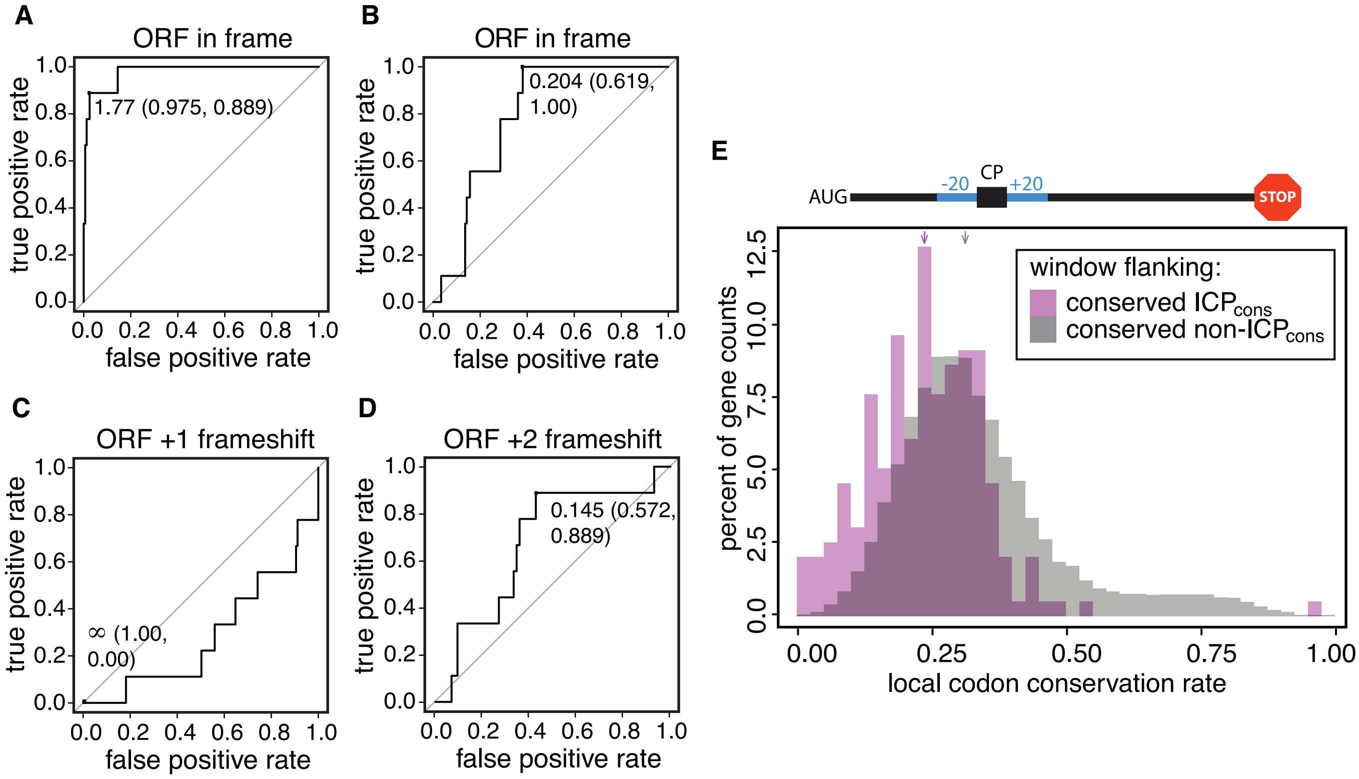

Figure S5 (refers to Figure 3). High conservation of the nine ICP$_{cons}$ is not due to the encoded dipeptide, sequence motifs, or their location in highly conserved sequences. *(A-D)*. *(A)* ICP$_{cons}$ in frame, *(B)* ACP in frame, *(C)* ICP$_{cons}$ +1 frameshift, *(D)* ICP$_{cons}$ +2 frameshift. Receiver-operator characteristic (ROC) curves, plotted using the z-score values as a classifier to discriminate each of the nine ICP$_{cons}$ from its synonymous codon pairs. ROC curves show the z-score with optimal specificity and sensitivity (shown in parenthesis). We found that a threshold of 1.77 σ from the mean distinguishes these ICP$_{cons}$ from other synonymous pairs with a specificity of 0.98 and a sensitivity of 0.89; the area under the curve is 0.90, indicative of an effective classifier. Thus, the high conservation of the ICP$_{cons}$ is still observed within the context of their dipeptide families. However, the threshold for the ROC curve for the ACPs is only 0.20 σ from the mean demonstrating that the conservation of ACPs is distinguishable from the conservation of other codon pairs for the same dipeptide, but with low specificity (0.62 for a sensitivity of 1.0). *(E)* Histograms of local codon conservation rates in the regions flanking conserved occurrences (-20 to +20 codons) of ICP$_{cons}$ (magenta) and other conserved codon pairs (gray). Arrows mark the median codon conservation scores (0.25 for ICP$_{cons}$ and 0.30 for other codon pairs).

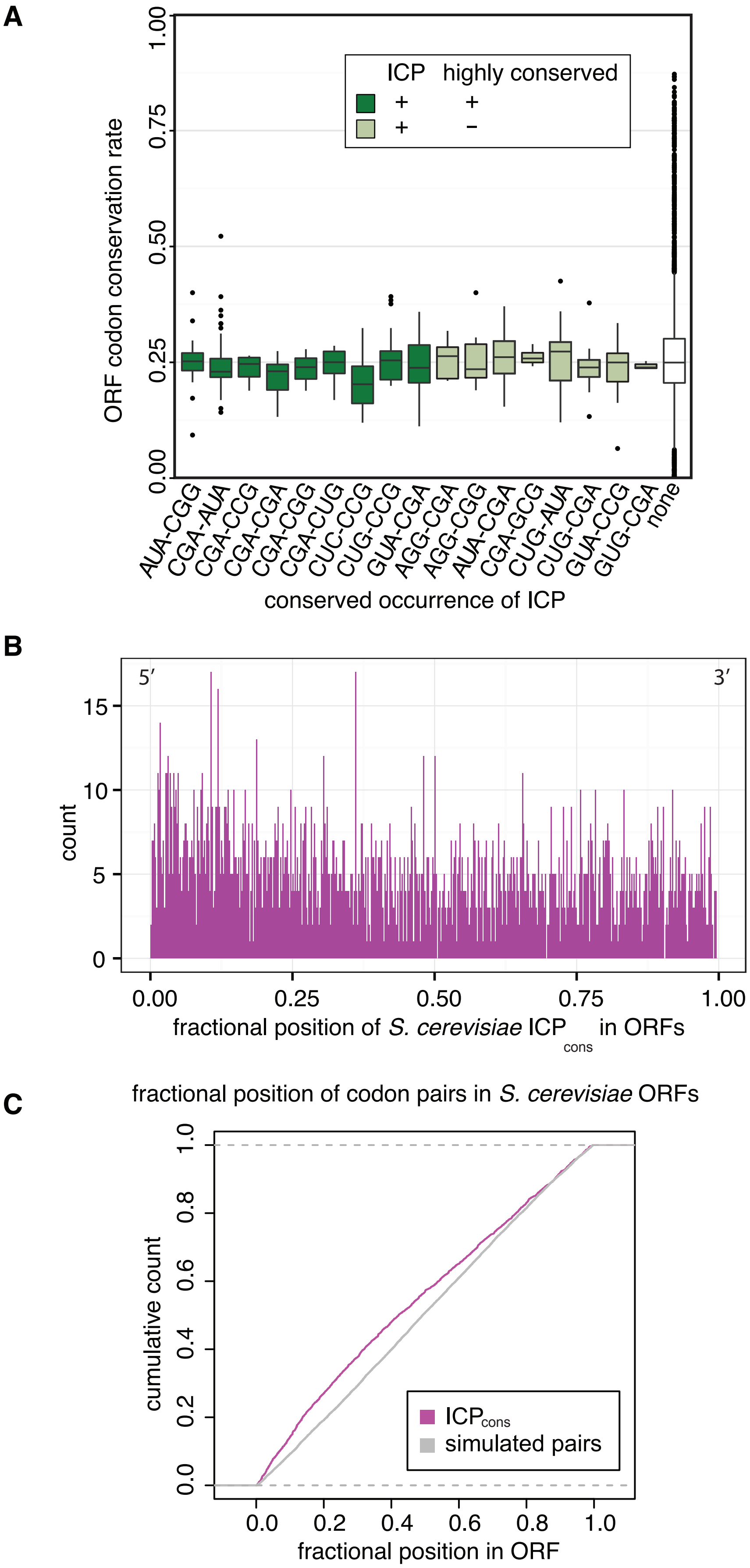

Figure S6 (refers to Figure 3). High conservation of ICP$_{cons}$ is independent of gene conservation, but is related to position in gene. *(A)* No individual ICP is associated with ORFs that are highly conserved. Box plot shows ORF codon conservation rates across genes containing a conserved occurrence of each of 17 known ICPs (ICP$_{cons}$ in dark green, other ICPs in light green) and genes not containing a conserved occurrence on any ICP (white). *(B)* Histogram of the relative positions of all ICP$_{cons}$ pairs in *S. cerevisiae* ORFs, with positions indicated as a fraction of the length of the ORF. *(C)* Cumulative distribution of relative position of all occurrences of ICP$_{cons}$ (magenta) in *S. cerevisiae* compared to occurrences of codon pairs in 100 simulations, each with nine randomly chosen codon pairs (gray).

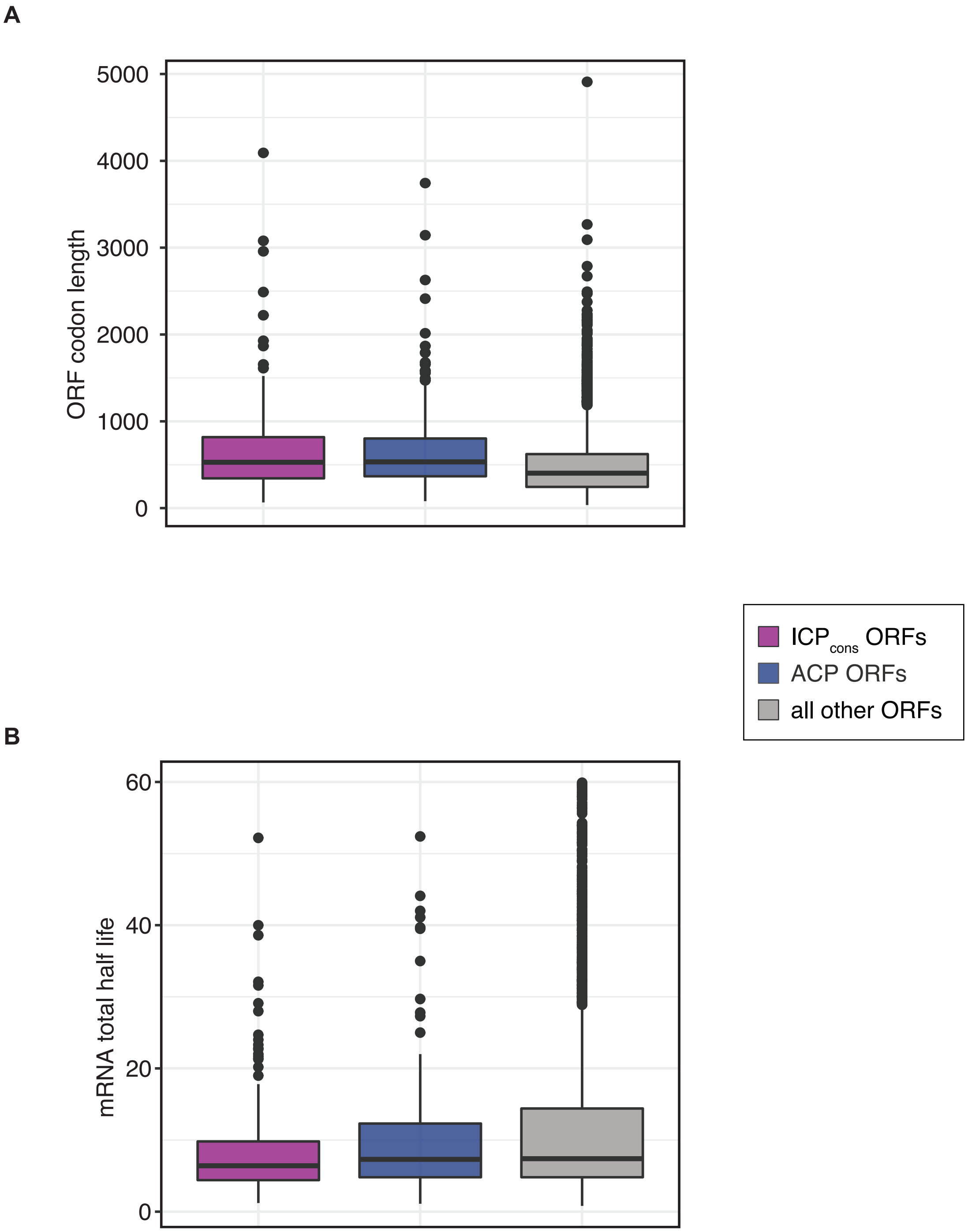

Figure S7 (refers to Table 1). Distribution of ORF lengths *(A)* and mRNA total half lives *(B)* are shown in boxplots for three gene sets: ICP_{cons} (magenta), ACPs (blue), and all other ORFs (gray).

Table S1. (refer to csv file) Analysis of codon and codon pair conservation across alignments from five *Saccharomyces sensu stricto* yeasts: *S. cerevisiae, S. paradoxus, S. kudriavzevii, S. mikatae, and S. bayanus v. uvarum*

Table S2. Inhibitory codon pairs and their corresponding alternative codon pairs (ACPs). Asterisks mark the subset of nine highly conserved ICPs (ICP_{cons}).

ICP	ICP dipeptide	Median syn-GFP^{SEQ}	ACP	ACP dipeptide	Median syn-GFP^{SEQ}
CGA-CCG*	RP	0.44	CCG-CGA	PR	1.00
CGA-CGA*	RR	0.44	CGC-CGA	RR	1.00
CGA-GCG*	RA	0.44	GCG-CGA	AR	0.98
CUC-CCG*	LP	0.44	CCG-CUC	PL	1.00
CGA-CUG	RL	0.47	CUG-CGC	LR	0.98
AGG-CGA	RR	0.48	CGA-AGG	RR	0.97
CGA-CGG*	RR	0.48	CGG-CGA	RR	0.91
CUG-CCG*	LP	0.49	CCG-CUG	PL	0.99
CUG-CGA	LR	0.50	CGA-CUU	RL	0.97
CGA-AUA*	RI	0.51	AUA-CAG	IQ	0.97
GUA-CGA*	VR	0.53	CGA-GUA	RV	0.98
AUA-CGA	IR	0.58	CAG-AUA	QI	1.00
GUG-CGA	VR	0.60	CGA-GUG	RV	0.99
AGG-CGG	RR	0.82	CGG-AGG	RR	0.98
AUA-CGG*	IR	0.65	CGG-AUA	RI	0.98
GUA-CCG	VP	0.80	CCG-GUA	PV	1.00
CUG-AUA	LI	0.71	AUA-CUG	IL	0.85

Table S3. (refer to csv file) Analysis of codon and codon pair conservation across pairwise alignments from *S. cerevisiae* and *S. paradoxus*

Table S4. (refer to csv file) Analysis of codon and codon pair conservation across pairwise alignments from *S. cerevisiae* and *S. kudriavzevii*

Table S5. (refer to csv file) Analysis of codon and codon pair conservation across pairwise alignments from *S. cerevisiae* and *S. mikatae*

Table S6. (refer to csv file) Analysis of codon and codon pair conservation across pairwise alignments from *S. cerevisiae* and *S. bayanus v. uvarum*

Table S7. (refer to csv file) Analysis of codon and codon pair conservation across pairwise alignments from *S. kudriavzevii*, and *S. mikatae*

Table S8. Conservation and rank of the nine highly conserved inhibitory codon pairs (ICP_{cons}) within their dipeptide families.

Codon Pair	Dipeptide	Synonymous pairs	Normalized codon pair conservation		Z-score	Codon pair conservation	
			Score	Rank[1]		Score	Rank[1]
AUA-CGG	IR	18	4.23	1	2.09	0.16	4
CGA-AUA	RI	18	3.99	1	1.84	0.30	1
CGA-CCG	RP	24	17.10	1	3.06	0.33	1
CGA-CGA	RR	36	13.59	1	2.35	0.57	1
CGA-CGG	RR	36	10.11	2	1.96	0.21	6
CGA-GCG	RA	24	15.60	1	2.48	0.19	1
CUC-CCG	LP	24	24.23	1	2.62	0.26	1
CUG-CCG	LP	24	17.22	2	2.27	0.11	2
GUA-CGA	VR	24	8.95	1	2.60	0.22	1

[1]Rank for each pair is the rank within its dipeptide family based upon codon pair conservation score (equation 2) and normalized codon pair conservation score (equation 3)

Table S9. (refer to csv file) Analysis of conservation in reading frame shifts across five *Saccharomyces sensu stritcto* yeasts: *S. cerevisiae, S. paradoxus, S. kudriavzevii, S. mikatae,* and *S. bayanus v. uvarum*

Table S10. (refer to csv file) Cumulative ribosome density (from Gamble *et al.*) and conservation of codon pairs

Table S11. (refer to csv file) List of ORFs containing an ICP_{cons} that is conserved across at least four species of *Saccharomyces sensu stricto* yeasts

Table S12. (refer to csv file) List of ORFs containing an alternative codon pair (ACP that is conserved across at least four species of *Saccharomyces sensu stricto* yeast.

Table S13. (refer to csv file) Summary of Gene Ontology category enrichment analysis of genes with ICP_{cons} conserved across at least four of the five *Saccharomyces sensu stricto* yeasts.

Table S14. (refer to csv file) GO biological processes categories enriched in genelist of ORFs containing an ICP_{cons} that is conserved across at least four species of *Saccharomyces sensu stricto* yeasts.

Table S15. (refer to csv file) GO cellular component categories enriched in genelist of ORFs containing an ICP_{cons} that is conserved across at least four species of *Saccharomyces sensu stricto* yeasts.

Table S16. (refer to csv file) GO molecular function categories enriched in genelist of ORFs containing an ICP_{cons} that is conserved across at least four species of *Saccharomyces* sensu stricto yeasts.

Table S17. (refer to csv file) GO biological processes categories enriched in genelist of ORFs containing an ACP that is conserved across at least four species of *Saccharomyces sensu stricto* yeasts.

Table S18. (refer to csv file) (refer to csv file) Analysis of codon and codon pair conservation across pairwise alignments from *Candida albicans* and *Candida dubliniensis.*
